# Team cohesiveness and collective efficacy explain outcomes in interprofessional education

**DOI:** 10.1186/s12909-022-03886-7

**Published:** 2022-11-29

**Authors:** Fraide A. Ganotice, Linda Chan, Xiaoai Shen, Angie Ho Yan Lam, Gloria Hoi Yan Wong, Rebecca Ka Wai Liu, George L. Tipoe

**Affiliations:** 1 Bau Institute of Medical and Health Sciences Education, the University of Hong Kong, Pokfulam, Hong Kong; 2 Li Ka Shing Faculty of Medicine, the University of Hong Kong, A5-15, 5/F, William MW Mong Block, 21 Sassoon Road, Pokfulam, Hong Kong; 3 School of Nursing, the University of Hong Kong, Pokfulam, Hong Kong; 4 Department of Social Work and Social Administration, the University of Hong Kong, Pokfulam, Hong Kong; 5 Li Ka Shing Faculty of Medicine, the University of Hong Kong, Rm L4-51, 4/F, Laboratory Block, 21 Sassoon Road, Pokfulam, Hong Kong

**Keywords:** Team cohesiveness, Collective efficacy, Team collaboration, Interprofessional education

## Abstract

**Background:**

Team cohesiveness and collective efficacy have been construed as important characteristics of a high-functioning team. However, the psychological mechanism through which they promote positive outcomes remains unknown. Understanding this psychological process is important to teachers and programme implementers to yield actionable interventions that can be used to craft effective practices for optimizing team outcomes. This is especially true in interprofessional education (IPE) in medical education, where a team-based approach to patient management is promoted. Drawing from the social-cognitive theory, we examined a hypothesized model where team cohesiveness predicts collaboration outcomes (teamwork satisfaction, overall satisfaction with the team experience, and IPE goal attainment) via collective efficacy.

**Methods:**

We used data from Chinese medicine, medicine, nursing, and social work students in Hong Kong (*n* = 285) who were enrolled in IPE. They were invited to respond to scales in two time points. We performed mediation analysis using structural equations modelling to test the indirect effect model: team cohesiveness → collective efficacy → outcomes.

**Results:**

Results of structural equation modelling revealed that collective efficacy fully mediated the relationships between team cohesiveness and all three team outcomes, providing support for the hypothesised model [RMSEA = 0.08, NFI = 0.90, CFI = 0.93, IFI = 0.93, TLI = 0.93]. Team cohesiveness predicted the achievement of collaboration outcomes via collective efficacy.

**Conclusion:**

The findings demonstrated the important roles of team cohesiveness and collective efficacy in promoting successful team collaboration. Team cohesiveness predicted collective efficacy, and collective efficacy, in turn, predicted collaboration outcomes. This study contributed to theorising the pathways towards successful team collaboration outcomes.

## Background

“Marvel’s The Avengers, featuring Iron Man, Captain America, the Hulk, and Thor is not just inspiring for comics fans.There's a huge lesson about teamwork you might not have paid attention to. Not just teamwork – but the value of teams themselves…When you're part of a team, a truly cohesive unit that functions with a single purpose, you can accomplish wonders.” [1].

Team performance is explained not just by a single factor but by various factors [2], including members’ belief of what the team can accomplish, as suggested by the vignette. In work or school settings, among the characteristics of desirable team members are being driven to contribute their best to achieve group goals, persisting in times of difficulties, and having confidence in their members’ abilities. Psychologists termed this collective efficacy (CE), which refers to a shared belief in the group’s capability to accomplish goals, which has been linked to key psychological outcomes, [3–5] including group performance [6, 7] Bandura, [8] (p477) further explained that CE “represents a group’s shared belief in its conjoint capabilities to organize and execute the courses of action required to produce given levels of attainments.“ It influences team members’ actions, the amount of effort they put into it, and their staying power when their efforts fail to produce the desired results [9]. CE is strongly linked with action since individuals in a group have little incentive to act unless they believe that their actions will produce the desired outcomes [10].

CE has been gaining traction in many areas, given its role in achieving important team goals. For example, research on CE has been implemented in various social systems: team sports, [7, 11–17] leadership attributes, [18–20] classroom learning, [21, 22] urban neighbourhoods, [23, 24] combat teams, [25] and political system [26]. In the context of healthcare, CE has likewise been linked with positive outcomes, such as reduced missed care, improved patient outcomes, [27] and improved nursing performance [28]. These findings provide support for the notion that CE can contribute to healthcare teams’ attainment of positive group outcomes.

However, despite the existence of literature establishing the cognitive [29] and affective consequences [30] of CE in teams, antecedent factors that promote CE are still relatively underexplored, especially in IPE. Model testing, which aims primarily at understanding IP teams, remains uncommon. Our understanding of the mechanism involved is paramount in informing us about the processes by which outcomes are achieved. We propose team cohesiveness as an antecedent to CE, which in turn, will predict academic-related outcomes (i.e., teamwork satisfaction, satisfaction with team experience, and goal attainment). Although previous studies established the link between collective efficacy and students’ of IPE learning outcomes [31, 32], to our knowledge, no study has examined team cohesiveness and its antecedents and outcomes in one analytic model. Examining this model can inform both theory and practice in IPE.

Moreover, there is a lack of studies on group processes in medical education. Despite the traction IPE is getting in medical education, the psychological mechanism that determines the achievement of key collaboration-related outcomes remains poorly understood. For instance, in medical education, where an interprofessional team-based approach is emphasised as a way to circumvent avoidable medical errors and provide patient-centred care, there is reason to believe that team cohesiveness can benefit the members of the healthcare team as they carry out their clinical duties, [33] through increased CE.

This study hopes to address the abovementioned limitations and extend the existing research in the following ways. *First*, we investigated the mediating role of CE in the relationship between team cohesiveness and teamwork satisfaction, overall satisfaction with team experience, and goal achievement. This study could explain the mechanism or underlying process in the relationships among these variables. *Second*, the study was conducted in the context of medical education. Specifically, we included interprofessional teams involving Chinese medicine, medicine, nursing, and social work students who performed care-based and team-based learning activities under the interprofessional education (IPE) learning module on depression. This study was in response to the call to approach IPE research with conceptual and methodological rigours through the integration of robust theories into interprofessional science [34, 35]. Lastly, the study was conducted in an Asian setting, which is relatively underexplored in the field. Hence, this study could provide useful information to help understand group processes in the context of IPE in medical education.

### Social-cognitive theory

To understand the role of CE in team cohesiveness and outcomes, we draw on the social-cognitive theory (SCT). SCT is a learning theory that focuses on the agentic perspective suggesting the ability of individuals to produce and shape experiences. It asserts that believing that one’s actions can produce desired outcomes could serve as an incentive to act or persevere when faced with difficulties [36]. In SCT, an individual’s cognitions are viewed as processes intervening between environmental stimuli and responses in real-world situations. We propose that the same applies to team members’ shared beliefs.

While the team members’ shared beliefs in their concerted ability to produce desirable results are the key driver of collective efficacy, the factors that drive CE appraisal, especially in IPE, are underexplored. We propose that this efficacy appraisal is influenced by team cohesiveness. That is, when team members are bonded to stay in the group (team cohesiveness), this will influence their CE appraisal, which will then affect team outcomes.

### Team cohesiveness and collective efficacy

Cohesiveness refers to the magnitude or strength of each team member’s intent to stay in the team, [37] which encompasses social and task forces that bring and keep individuals together [38]. The literature describes team or group cohesiveness as the members’ attraction and bond to one another. Team cohesiveness drives members to develop conformity and remain part of the team despite the challenges [39, 40].

Previous studies identified a number of antecedents to collective efficacy, including leadership, [41, 42] motivational climate, [43] team cohesion, [44–46] previous expectation, [47] and past performance, and group size [48]. These factors related to team cohesiveness could influence members’ appraisal of CE levels. In team sports, empirical studies have examined the relationships between cohesiveness, collective efficacy, and performance [42]. Furthermore, the work of Zaccaro et al. [49] suggests that cohesion can contribute to teams’ efficacy appraisal.

While most studies examined the direct relationship of team cohesiveness with outcomes, the studies above suggest the potential mediating role of collective efficacy. Despite the evidence demonstrating the links between CE and adaptive outcomes, very few have examined the relationships among team cohesiveness, collective efficacy, and positive outcomes (e.g., Jung and Sosik’s study [50] involving the leadership of Korean firms). Moreover, to the best of our knowledge, none have explored this model in the context of IPE.

### Collective efficacy and team outcomes

The link between CE and team outcomes has been widely supported in theory and empirical studies. For example, in the classroom setting, Zander [51] found between-group differences when high and low efficacy groups were compared. High efficacy groups outperformed the low efficacy groups in terms of goal achievement [52]. Similarly, high efficacy groups were found to set more challenging goals and were more determined to achieve those goals [53].

CE has also been found to have a unique effect on teachers’ job satisfaction; [54] as well as satisfaction with one’s own performance and team performance in sports [55]. Given the strong link between CE and group outcomes, we propose that CE in interprofessional healthcare teams would predict collaboration outcomes: teamwork satisfaction, overall satisfaction with team experience, and IPE goal achievement.

### Context of the study

**Fig. 1:**
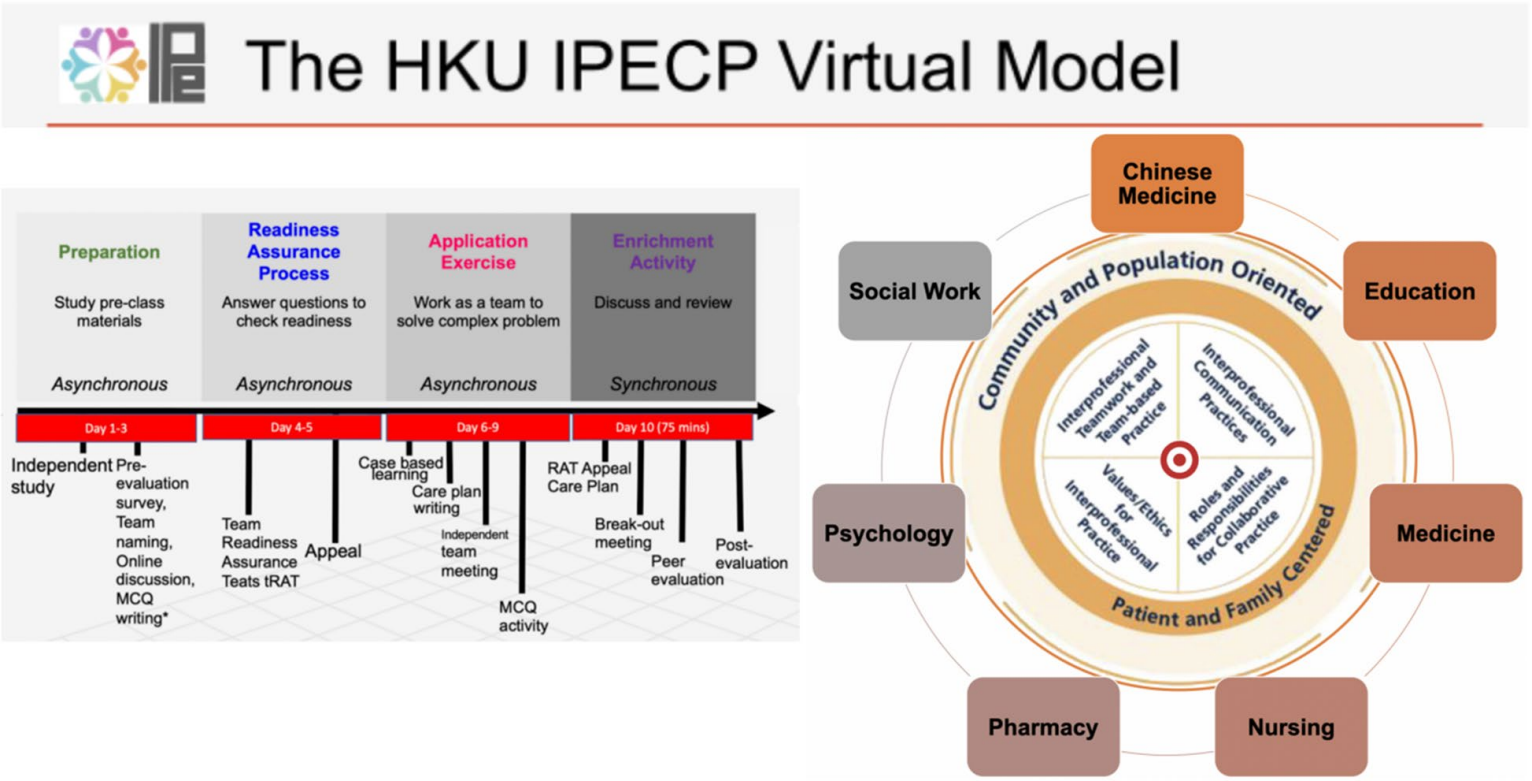
The ten-day interprofessional education model

The current study was conducted on a 10-day IPE asynchronous and synchronous programme in a government-subsidised learning institution in Hong Kong. As it was carried out during the height of the COVID-19 pandemic, the programme was administered using online tools to facilitate team interactions. These tools included digital chat/discussion/whiteboard for optimal interaction, share screen, main room, and break-out room functions of Zoom for team meetings and presentations, Google slides for interprofessional management care planning, and Kahoot and Mentimeter to gamify the synchronous sessions [56]. The IPE healthcare teams were composed of students from Chinese medicine, medicine, nursing, and social work. Figure 1 describes the IPE model of the programme through which this study was conducted.

**Fig. 2:**
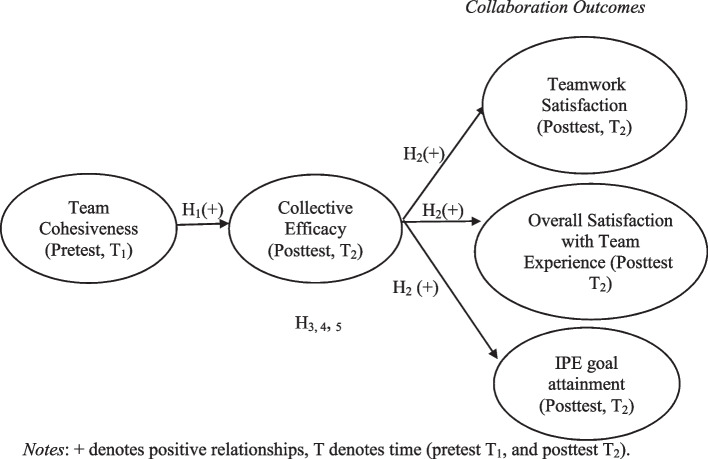
The hypothesized model of the relationships between cohesiveness, efficacy, and collaboration outcomes

### The present study

This study aims to understand the psychological mechanism and team processes that underlie positive team outcomes in medical education. Specifically, we intend to examine the mediational effect of team cohesiveness, collective efficacy, and team outcomes (see Fig. 2). Although CE has been found to have a proximal effect on outcomes, and while there are also studies that explored its antecedents, examining such relations in a unified analytic model remains unexplored, especially in the context of IPE in medical education. Hence, we propose that CE can mediate the relationship between team cohesiveness and team outcomes. The following are the specific hypotheses tested in the study:

H_1_: Team cohesiveness predicts collective efficacy.

H_2_: Collective efficacy predicts collaboration outcomes (teamwork satisfaction, overall satisfaction with team experience, and IPE goal attainment).

H_3_: Collective efficacy mediates the relationship between team cohesiveness and teamwork satisfaction.

H_4_: Collective efficacy mediates the relationship between team cohesiveness and overall satisfaction with team experience.

H_5_: Collective efficacy mediates the relationship between team cohesiveness and IPE goal attainment.

The ability of CE to foster team outcomes may induce the likelihood of cultivating better academic engagement and achievement in IPE. The study was conducted in the context of IPE in Hong Kong.

## Method

### Participants

The participants were 285 students (undergraduate level = 227; master’s level = 58) from a government subsidized learning institution in Hong Kong. They were from prelicensure programmes in Chinese medicine (*n* = 20), medicine (*n* = 96), nursing (*n* = 90), social work (*n* = 21), and master’s programme in social work (*n* = 58). In mid-February 2020, when all measures were administered, the mean age of participants was 22.42 years (*SD* = 2.18). Of the 285 participants, 7% were in Year III, 72.6% were in Year IV, and 20.4% were in the master’s programme; 56% were female, while 44% were males. The participants were all required to participate in a cross-faculty and cross-programme ten-day asynchronous and synchronous IPE programme. However, their involvement in the pre- and post-evaluation survey was entirely voluntary. The Human Research Ethics Committee (HREC, EA1507012) granted ethics approval for this study. Participants gave us their informed consent.

### Measures and procedures

We used the following scales to measure the study variables: team cohesiveness, collective efficacy, team satisfaction, overall satisfaction with team experience, and IPE goal achievement. The measures were adapted/validated to fit within the IPE context.

#### Team cohesiveness

We used the 4-item scale of Seashore [57]. For instance, we specified the term “IPE team” to make the items specific to the IPE programme context (e.g., “*We got along with others as IPE team.“)*. We performed confirmatory factor analysis (CFA) to test the goodness of fit of the scale. The analysis showed it has good fit [χ^2^ = 298.19, *df* = 35 (303.30), NFI = 0.93, IFI = 0.93, CFI = 0.93, and RMSEA = 0.06].

#### Collective efficacy

We used the 4-item Generalized Self-efficacy Assessment [58] We adapted the items in the context of work groups, which was similar to the procedure employed by Salanova et al. [59]. For example, we modified the item “*I am totally competent to solve the task*.“ To “*My IPE team is totally competent to solve the task*.“ The response scale ranged from 1 (never) to 5 (most of the time). CFA results indicate the model has good fit [χ^2^ = 23.079, *df* = 2 (11.53), NFI = 0.98, IFI = 0.98, CFI = 0.98, and RMSEA = 0.08].

#### Teamwork satisfaction

We used the Online Teamwork Satisfaction Scale developed by Tseng et al. [60]. The instrument was composed of 10 items, rated on a five-point Likert scale ranging from 1 (*strongly disagree*) to 5 (*strongly agree*). The CFA performed using the current data yielded a good fit [χ^2^ = 23.079, *df* = 2 (11.53), NFI = 0.98, IFI = 0.98, CFI = 0.98, and RMSEA = 0.08].

#### Overall satisfaction with team experience

We used the subscale overall satisfaction with team experience from the Students’ Team Experience Questionnaire [61]. This subscale is composed of five items (e.g., “*I have found working as part of an IPE team in my classes to be a valuable experience*.“), with a response scale ranging from 1 (*strongly disagree*) to 5 (*strongly agree*). The CFA performed using the current data yielded good fit [χ^2^ = 5.726, *df* = 2 (2.86), NFI = 0.99, IFI = 0.99, CFI = 0.99, and RMSEA = 0.08].

#### IPE goal attainment

We used the 7-item perception of attainment in IPE learning outcomes (e.g., “*collaborate with students in other professions in solving clinical problems*”). Students were instructed to assess the extent of attainment of the IPE learning outcomes and rank the items from 1 (*to the least extent*) to 5 (*to the greatest extent*). We particularly provided this instruction to the participants: “*Please indicate your perception of the extent of attainment of the following objectives as a result of your involvement in interprofessional education*.“ CFA results demonstrated a good model fit [χ^2^ = 5.726, *df* = 2 (2.86), NFI = 0.99, IFI = 0.99, CFI = 0.99, and RMSEA = 0.08].

To examine the programme effects, we collected the data at two time points. Specifically, we collected the data on team cohesiveness halfway through the programme, on the fifth day of the ten-day asynchronous and synchronous IPE (Time 1). We collected the data on collective efficacy, teamwork satisfaction, overall satisfaction with team experience, and IPE goal achievement on the last day as part of the posttest (Time 2). The students were invited to complete the questionnaires after the synchronous session. However, their participation in the study was voluntary.

### Data analysis

To test the proposed model of team cohesiveness predicting team outcomes (teamwork satisfaction, overall satisfaction with team experience, and IPE goal achievement) through collective efficacy, we conducted structural equation modelling (SEM). Team cohesiveness was posited as the distal antecedent, CE as the mediator, and team outcomes as the outcome variables (see Fig. 2). All the variables were designated as latent constructs underpinned by their corresponding manifest variables (the items). CFAs were conducted to examine the measurement validity of the scales.

We used AMOS 26.0 for the main analysis. We used the suggestions of Finney and DiStefano [62] regarding skewness and kurtosis where values beyond 2 and 7, respectively, suggest the lack of univariate normality. The skewness of our data ranged from − 0.80 to 1.46, while the kurtosis ranged from 0.88 to 3.81 for all items, indicating normality.

We used maximum likelihood as the method of estimation. To examine model fit, we used a number of goodness-of-fit indices: normed-fit index (NFI), comparative fit index (CFI), incremental fit index (IFI), Tucker-Lewis index (TLI), root mean square error of approximation (RMSEA), Akaike information criterion (AIC), and Bayes information criterion (BIC) [63].  For AIC and BIC, lower values are preferred when comparing the two models [64] In addition, for NFI, CFI, IFI, and TLI, values must be greater than 0.90, while the RMSEA value should be equal to or less than 0.08. We used bootstrapping procedures [65] to test the statistical significance of the indirect effect of the proposed model.

## Results

### Mediational analysis

We performed a mediation analysis to test the hypothesized model shown in Fig. 2. In this model, team cohesiveness (collected at Time 1) served as the predictor of the three collaboration outcomes: teamwork satisfaction, overall satisfaction with team experience, and IPE goal attainment (collected at Time 2). CE (also collected at Time 2) was posited as the mediator. Results showed that the data fit the model well [χ^2^ = (1150.54, *df* = 372) = 3.09, NFI = 0.90, CFI = 0.93, IIFI = 0.93, TLI = 0.93, and RMSEA = 0.08, see Table 1].

**Table 1:** The goodness of fit indices for the models

Model	χ^2^	df	*p*	χ^2^/df	RMSEA	NFI	CFI	IFI	TLI	AIC	BIC
Hypothesised model (team cohesiveness →collective efficacy→ outcomes)	1150.54	372	<.001	3.09	.08	.90	.93	.93	.93	1276.54	1506.65
Alternative model	1458.95	370	<.001	3.94	.10	.88	.90	.90	.89	1588.95	1826.36

*Notes*: The alternative model posits both team cohesiveness and collective efficacy as predictors of teamwork satisfaction, overall satisfaction with team experience, and IPE goal attainment. *RMSEA* Root mean square error of approximation, *NFI* Normed fit index, *CFI* Comparative fit index, *IFI* Incremental fit index, *TLI* Tucker–Lewis index, *AGFI* Adjusted goodness of fit index, *AIC* Akaike information criterion, *BIC* Bayes information criterion

Results showed that CE was positively associated with the three collaboration outcomes providing support to H_2_. Team cohesiveness was also positively associated with CE providing support to H_1_ (see Table 2). The direct relationship of team cohesiveness with the three measures of collaboration outcomes was not significant, suggesting full mediation providing support to H_3,4,5_ (see Table 2).

**Table 2:** Hypotheses and results

Hypotheses						Results
H_1_:	Team cohesiveness	→	collective efficacy			Confirmed
H_2_:	Collective efficacy	→	collaboration outcomes: teamwork satisfaction, overall satisfaction with team experiences, and IPE goal attainment			Confirmed
H_3_:	Team cohesiveness	→	collective efficacy	→	online teamwork satisfaction	Confirmed
H_4_:	Team cohesiveness	→	collective efficacy	→	overall satisfaction with team experience.	Confirmed
H_5_:	Team cohesiveness	→	collective efficacy	→	IPE goal attainment	Confirmed

## Discussion

The study aimed to understand the underlying psychological group processes or mechanisms that explain team outcomes in IPE in the context of medical education. Specifically, we tested a model of CE mediating the relationship between team cohesiveness and team outcomes (teamwork satisfaction, overall satisfaction with team experience, and IPE goal attainment). The findings of the study provided general support for SCT and demonstrated how this theory could be extended in the context of IPE among healthcare and social care Chinese students in Hong Kong.

The findings of the study indicated that team cohesiveness predicted collective efficacy, and collective efficacy, in turn, predicted the three collaboration outcomes, namely: teamwork satisfaction, overall satisfaction with team experience, and IPE goal attainment. Consistent with the widely established relationship between CE and positive outcomes due to collective efficacy’s role in effort sustenance, 8 the findings of the study provided support for the applicability of the theory in the context of IPE, as CE was found to promote collaboration outcomes. These findings are within the expected direction posited by SCT.

A noteworthy result of this study relates to the mediation analyses, which showed that a significant amount of variance in the collaboration outcomes could be attributed to the indirect effects of team cohesiveness via collective efficacy. That is, more cohesive IPE teams tend to have greater collective efficacy, which subsequently predicts increased teamwork satisfaction, overall satisfaction with team experience, and IPE goal attainment.

Another salient finding of the study that can contribute to the extant literature is, from a measurement perspective, the validity of the measures can be extended in the context of IPE in medical education. This study provides support for the utilisation of the measures of psychological processes in healthcare teams vis-à-vis team outcomes. This study has established the psychometric applicability of team cohesiveness, collective efficacy, team satisfaction, overall satisfaction with team experience, and IPE goal attainment in IPE in medical education involving Hong Kong Chinese learners. Examining the psychometric properties of constructs when proposed, adapted, and operationalised in a new context is indeed important [66].

From a practical viewpoint, we can draw important intervention implications from the findings, which are especially useful to IPE program implementers. The full mediating role of CE on the relations between team cohesiveness and IPE outcomes suggests that programme designers need to target the development of teams’ positive shared belief in their conjoint ability appraisal for their team to attain positive team outcomes, and a way to do that is to strengthen team cohesiveness. There are numerous ways to optimize CE in the context of IPE. For example, when teams are given authentic and challenging activities (e.g., discuss the readiness assurance test, formulate interprofessional healthcare management plan, debrief team interactions on completed activities), these can provide an opportunity for the team to strengthen their cohesiveness by working together. As a result, ability appraisal opportunities of what the team members from diverse disciplines (e.g., medicine, nursing, social work) can contribute to interprofessional team-based management of patients are induced. Further, a spiral approach to IPE delivery in which students are given various opportunities to develop team cohesiveness is worth considering. This study extends the findings of Egenberg et al. [32] on teams’ CE following interprofessional simulation training.

Another implication relates to the importance of SCT as a framework for understanding factors affecting the achievement of teams in IPE, especially in the Hong Kong Chinese context. This study extends the SCT further by demonstrating that social agency (e.g., teams and programme coordinators) can demonstrate supporting behaviours within which they can provide a context where team cohesiveness is nurtured, which can promote a favourable CE appraisal, leading to the attainment of positive collaboration outcomes. Our experience and empirical data in implementing a large-scale IPE indicate that well-designed team activities could promote the attainment of desirable outcomes through collective efficacy.

The strengths of this study notwithstanding, we also note some limitations and suggestions for future research. First, this study was based on self-reports, which are known to be influenced by both social desirability and common method variance. Second, we only considered a single antecedent of CE (i.e., team cohesiveness). We suggest that other researchers explore variables that may further enrich our understanding of the antecedents of collective efficacy. Third, the teams involved in this investigation were formed only for a ten-day simulation period. These teams were not established teams for the whole semester. Despite these limitations, the study has important contributions to theorising the pathways towards successful team collaboration outcomes in the IPE programme in Hong Kong.

We wish to underscore that this study contributes to filling the knowledge gap in the IPE literature about the influences of the team’s psychological processes on outcomes which can be developed through a brief IPE programme. Specifically, the study demonstrated that team cohesiveness could predict an increase in collective efficacy, which could subsequently predict positive IPE team outcomes.

## Data Availability

The dataset is available from the corresponding author upon reasonable request.
